# Haemophilia gene therapy: experiences and lessons from treated patients

**DOI:** 10.1186/s13023-022-02313-w

**Published:** 2022-04-04

**Authors:** Cedric Hermans

**Affiliations:** Division of Adult Hematology, Haemostasis and Thrombosis Unit, Cliniques universitaires Saint-Luc, Université catholique de Louvain (UCLouvain), Avenue Hippocrate 10, 1200 Brussels, Belgium

**Keywords:** Haemophilia, Gene therapy, Decision making, Informed consent, Clinical trial

Two decades of basic research and several recent clinical trials have turned the long-awaited hope of gene therapy for haemophilia into a reality [1–3]. The principle is to endow liver cells with the ability to produce clotting factor VIII (FVIII) or IX (FIX), whose genetically induced defect in synthesis characterises haemophilia A and B respectively. The aim is to induce sufficient endogenous production of these clotting factors in the long term, thereby ensuring that no haemorrhages occur, particularly in the joints. For persons with haemophilia, the main expected benefit is the release of the treatment constraint. The latter consists of regular, burdensome intravenous injections of FVIII or FIX concentrates or subcutaneous administrations of agents that mimic the action of FVIII or modify the balance of blood coagulation [4].

The technology uses adeno-associated viral (AAV) vectors with a hepatic tropism. After a single intravenous administration, these vectors transport the FVIII and FIX genes to the liver. After internalisation by the hepatocytes, these genes, which do not integrate into the genome, are transcribed into FVIII and FIX molecules and released into the bloodstream [1].

The approach is very attractive but not without obstacles and challenges. The first is the presence in many gene therapy candidates of neutralising antibodies directed against the capsid of viral vectors resulting from previous exposure to these almost ubiquitous viruses. The second is the occurrence of an immune reaction directed against the transfected hepatocytes, which may result in the loss of the latter. Strict biological monitoring of liver integrity is essential during treatment, as is the rapid introduction of immunosuppressive therapy to control rejection reactions.

Despite these difficulties, gene therapy for haemophilia is now possible as demonstrated by the results of several ongoing or completed clinical trials [5–7]. These trials show that after gene therapy, people with haemophilia can achieve normal levels of FVIII or FIX, no longer develop bleeding complications and not require replacement therapy.

Behind these exciting results, however, lie other realities of gene therapy. The production of FVIII or FIX among treated people is highly variable and unpredictable, rarely absent and sometimes supranormal. Most patients require immunosuppressive treatment, which can be complex and prolonged and is not without side effects. Factor production seems to decrease over time, at least for FVIII, so that it is difficult to predict how long the effect of the treatment will last.

Gene therapy for haemophilia is therefore as attractive as it is confusing, as exciting as it is worrying. In this context, it is critical to take into account the expectations of persons with haemophilia who are potential candidates for gene therapy. Some studies have recently explored this through interviews [8, 9]. Other studies have tried to optimize communication of gene therapy [10] or determine which factors promote or hinder the adoption of gene therapy and guide the selection of candidates [11]. In addition to the current classic exclusion criteria such as, among others, age, the presence of comorbidities and active liver diseases, many other factors could be involved, often subjective and personal, which prevent many patients from considering gene therapy.

In addition to the objective clinical and biological results of recent clinical trials, the experiences of patients treated with gene therapy should be surveyed and shared with the community. This is the merit of this article reporting the results of the Exigency Study conducted by Simon Fletcher and colleagues. In this original study, 16 patients (11 HA and 5 HB) who took part in clinical trials from the UK, 10 accompanied by a family member, provide valuable information of their experience of gene therapy [12].

Many patients, especially those who participated in the Phase 1 studies, explained that altruism and concern for helping future generations motivated them. For others, the main reason was the possibility of a cure for their haemophilia. Thirteen patients required immunosuppressive (IS) treatment. Ten of them report IS being the worst part of their experience with gene therapy. Many reported the strong pressure from health professionals to continue IS treatment even in the presence of side effects. The loss of control and individuality required by the study protocols, including biological monitoring and the need for IS, are other important observations. Several participants mentioned that their mental health and psychological difficulties were not sufficiently taken into account. Despite these constraints, this study confirms that gene therapy is perceived and experienced as a real liberation by most patients.

Some weaknesses and limitations of this study cannot be denied. The study population was limited but it represents 50% of those with hemophilia in UK who have had gene therapy and is currently a larger cohort than any other yet published. Other potential limitations are a selection bias of participants and the assumption that some experiences and opinions might not have been explored. The rigorous methodology and approach of the study very likely contributed to capture reliably most of the current knowledge, expectations and experiences among patients treated with gene therapy. The study was conducted in a developed country providing excellent access to haemophilia treatment and care. The issues and concerns raised may likely differ from those of low- and middle-income countries.

These limitations cannot obscure the merits of the study in having surveyed by independent interviewers unconnected with haemophilia centres and pharmaceutical companies the experience and background of a relevant number of patients treated with gene therapy and their family members.

This study underlines the importance of education and transparent information to candidates for gene therapy and their families about the modalities of this revolutionary treatment, its constraints and uncertainties [13]. The same applies to the challenges of immunosuppression, which are often poorly accepted and tolerated as well as the need for psychological support.

These steps are the responsibility of all partners involved in haemophilia gene therapy, whether they are health professionals, patient associations or pharmaceutical partners. Beyond biological (FVIII or FIX levels after gene therapy) and clinical (number of bleeds) results, it is important to be able to share with potential future candidates high-quality real-life data reflecting the personal experiences and perceptions of patients already treated. This article, whose content can be easily understood even by non-specialists and patients, should contribute to this objective and motivate other comparable initiatives in the future when gene therapy will be accessible outside clinical trials.
